# ATR-CHK1 Axis Inhibitors in Gastric Cancer Treatment

**DOI:** 10.3390/ijms26167709

**Published:** 2025-08-09

**Authors:** Mateusz Kciuk, Renata Gruszka, Marta Aleksandrowicz, Agnieszka Śliwińska, Renata Kontek

**Affiliations:** 1 Department of Molecular Biotechnology and Genetics, Faculty of Biology and Environmental Protection, University of Lodz, Banacha Street 12/16, 90-237 Lodz, Poland; renata.gruszka@biol.uni.lodz.pl (R.G.); renata.kontek@biol.uni.lodz.pl (R.K.); 2 Laboratory of Preclinical Research and Environmental Agents, Mossakowski Medical Research Institute, Polish Academy of Sciences, 5 A. Pawińskiego Street, 02-106 Warsaw, Poland; maleksandrowicz@imdik.pan.pl; 3 Department of Nucleic Acid Biochemistry, Medical University of Lodz, Pomorska 251, 92-213 Lodz, Poland; agnieszka.sliwinska@umed.lodz.pl

**Keywords:** gastric cancer, ATR–CHK1 inhibitors, DNA damage response, replication stress, synthetic lethality, TP53 mutation, *ARID1A* loss, immune modulation, treatment resistance, biomarker-guided therapy

## Abstract

Gastric cancer remains a significant global health challenge, with regional and demographic disparities in incidence, mortality, and treatment outcomes. Despite advances in screening and early detection, prognosis remains poor for many patients, particularly those with advanced disease. Recent insights into DNA damage response pathways have uncovered critical molecular vulnerabilities in gastric tumors, including frequent *TP53* mutations, *ARID1A* loss, *ATM* deficiency, and oncogene-driven replication stress, which render these cancers highly dependent on the ATR–CHK1 axis for survival. This review synthesizes current clinical and preclinical evidence on ATR and CHK1 inhibitors as therapeutic strategies in gastric cancer. Emphasis is placed on synthetic lethality, immune modulation, and the potential for combination regimens with chemotherapy, radiotherapy, or immune checkpoint blockade. Mechanisms of resistance, including transcription-associated replication stress modulation and bypass signaling networks, are discussed, alongside strategies to predict and overcome therapeutic failure. The review also highlights the importance of biomarker-guided patient selection, adaptive dosing to reduce toxicity, and refined pharmacodynamic monitoring to enhance therapeutic precision. Collectively, these insights support the rational integration of ATR–CHK1 inhibitors into clinical protocols for biomarker-defined gastric cancer subsets and underscore their promise

## 1. Introduction

**Figure 1:**
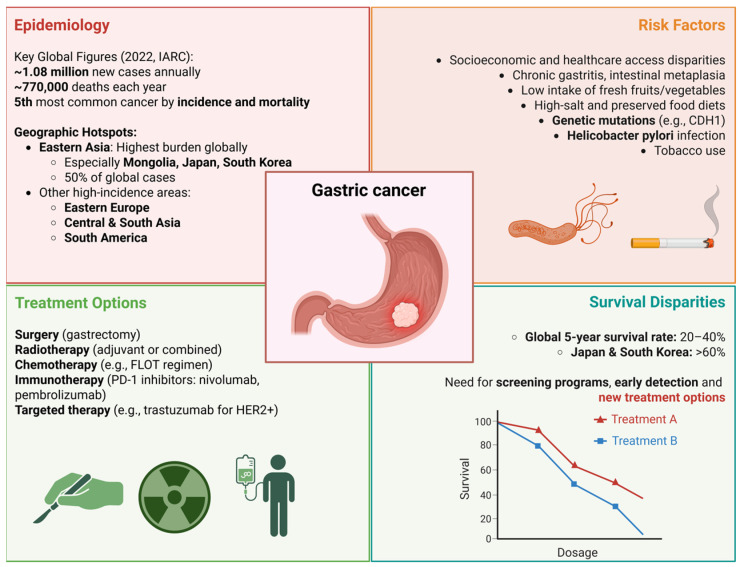
Gastric cancer epidemiology, risk factors, treatment options associated with survival benefits. CDH1—cadherin 1; FLOT—fluorouracil/leucovorin, oxaliplatin, and docetaxel; HER2—human epidermal growth factor receptor 2. Created in BioRender. Kciuk, M. (2025) https://BioRender.com/de9buio (accessed on 8 August 2025).

Gastric cancer remains a major global health burden, although its epidemiological landscape has shifted significantly over recent decades. Once the second most common cancer worldwide in 1990, gastric cancer ranked fifth in both incidence and mortality in 2022, according to estimates by the International Agency for Research on Cancer (IARC). Despite a steady overall decline in incidence and mortality, the disease still accounts for over 1 million new cases and nearly 770,000 deaths annually, underscoring its persistent global impact. A defining feature of gastric cancer epidemiology is its marked geographical variation. Eastern Asia continues to bear the highest burden, particularly countries like Mongolia, Japan, and South Korea, which collectively account for more than half of global cases. Other high-incidence regions include Eastern Europe, Central and South Asia, and parts of South America. Age-standardized rates in Eastern Asia far exceed those in other parts of the world [1,2,3], emphasizing the importance of regional risk factors such as *Helicobacter pylori* infection [4,5,6,7], dietary patterns [8,9,10,11], and screening practices [12,13,14]. Survival outcomes also vary widely by region. Data from the CONCORD-3 study reveal that while global five-year relative survival rates for gastric cancer generally range between 20 and 40%, Japan and South Korea have achieved much higher rates, over 60%, largely due to national screening programs and early detection initiatives [15]. Although overall trends suggest declining incidence and mortality, recent analyses indicate that these improvements are not universal. Join-point regression analysis reveals that several countries, including Thailand, Malta, and Canada, have experienced rising incidence or mortality trends, particularly among younger populations under 45 years of age. Projections to 2035 suggest that while age-adjusted mortality rates may continue to fall, the absolute number of deaths could rise in certain populations due to demographic shifts, especially in countries such as France, Israel, and Chile [3]. These evolving patterns highlight the need for continued surveillance, targeted prevention strategies, and health policy interventions tailored to regional and demographic risk profiles. Figure 1 displays epidemiological data and treatment options for gastric cancer.

As indicated previously, the global incidence and mortality rates of gastric cancer have shown a marked decline. This trend is largely attributed to a combination of medical, environmental, and socioeconomic factors. A primary contributor is the significant reduction in *Helicobacter pylori* infection rates, a well-established risk factor for non-cardia gastric cancer. This decline is likely a result of improved sanitation [16,17], widespread antibiotic use [18,19,20,21], and heightened public health awareness [22,23,24,25]. Concurrently, dietary patterns have shifted away from traditional carcinogenic food preservation methods, such as smoking, salting, and pickling, toward increased consumption of fresh fruits and vegetables, facilitated by the widespread adoption of refrigeration [25]. Tobacco control efforts have also contributed, as smoking cessation is associated with reduced gastric cancer risk [26,27,28,29]. In addition, advances in diagnostic endoscopy [30,31,32,33,34] and the implementation of screening programs in certain high-incidence regions have enabled earlier detection and removal of premalignant lesions and localized tumors [12,13,14].

Importantly, treatment strategies for gastric cancer have also evolved substantially. In the medical oncology domain, perioperative chemotherapy has become the standard of care for locally advanced resectable disease, exemplified by regimens such as FLOT (fluorouracil, leucovorin, oxaliplatin, and docetaxel), which have demonstrated superior survival benefits compared to previous protocols [35,36,37]. For metastatic disease, systemic therapy options have expanded significantly. Platinum- and fluoropyrimidine-based combinations remain foundational, but biomarker-driven approaches are increasingly integrated into treatment algorithms. Targeted therapies such as trastuzumab for human epidermal growth factor receptor 2 (HER2)-positive tumors and ramucirumab, a vascular endothelial growth factor receptor 2 (VEGFR-2) inhibitor, have improved outcomes in selected patients [38,39]. More recently, the incorporation of immune checkpoint inhibitors, such as nivolumab and pembrolizumab, has demonstrated clinical benefit in subgroups defined by programmed death-ligand 1 (PD-L1) expression, microsatellite instability (MSI), or high tumor mutational burden [40,41,42,43].

The advent of DNA damage response (DDR) inhibitors has significantly transformed the landscape of cancer therapy by introducing a new paradigm in precision oncology. Traditionally, cancer treatments such as chemotherapy and radiation targeted rapidly dividing cells, including cancerous ones, but often at the cost of considerable toxicity to normal tissues [38,39]. The cellular response to chemotherapy and radiotherapy involves two interrelated but distinct processes: the DDR and the repair of DNA damage. The DDR encompasses a complex signaling network that detects DNA lesions, transduces signals through kinase cascades, and orchestrates downstream cellular outcomes such as cell cycle arrest, transcriptional reprogramming, senescence, or apoptosis [44,45,46]. In contrast, DNA repair refers specifically to the enzymatic pathways that directly remove or correct damaged DNA [47,48,49]. While the DDR facilitates the recruitment and regulation of these repair machineries, it is not synonymous with repair itself. Rather, it represents a broader cellular surveillance mechanism that governs both the detection of damage and the decision-making process for appropriate cellular outcomes based on the severity and context of the lesions.

DDR inhibitors exploit the inherent genetic vulnerabilities of cancer cells, specifically, their compromised ability to repair DNA damage, offering a more selective and effective therapeutic approach [50,51]. A key concept in DDR inhibitor development is synthetic lethality. The idea is that while cells can survive the loss of one DNA repair pathway, simultaneous inhibition of two leads to cell death [52,53,54,55]. This concept is exemplified by the success of poly ADP-ribose polymerase (PARP) inhibitors in tumors harboring breast cancer type 1 susceptibility protein 1/2 (*BRCA1*/*BRCA2*) mutations. The success of PARP inhibitors has spurred the development of inhibitors targeting other DDR proteins, including ataxia telangiectasia and Rad3-related (ATR) kinase, checkpoint kinases (CHK1/2), and others [45,56,57]. These agents are being explored as monotherapies or in combination with chemotherapy, radiotherapy, or immunotherapy to enhance tumor-specific cytotoxicity and overcome resistance mechanisms [58,59].

In the context of gastric cancer, several studies have emphasized the crucial role of DDR pathways in the pathogenesis and progression of gastric cancer, highlighting several molecular markers with diagnostic, prognostic, and therapeutic relevance as recently reviewed by Wang M. and Xie C. [60] Here, we aimed to review clinical and emerging pre-clinical evidence on the role of ATR-CHK1 axis inhibitors in gastric cancer therapy.

## 2. ATR/CHK1 Axis in DDR

DNA replication stress (RS) represents a central mechanism in the initiation and progression of tumorigenesis, particularly through its role in driving genomic instability, a hallmark of cancer [61,62]. During each cell cycle, the replication fork encounters numerous exogenous and endogenous barriers that compromise replication fidelity and fork progression. External stressors such as ultraviolet (UV) light, ionizing radiation, and DNA-damaging chemotherapeutics (e.g., alkylating or cross-linking agents) generate lesions that physically obstruct replication machinery. Simultaneously, internal sources of RS, including reactive oxygen species (ROS), metabolic byproducts, such as aldehydes, and aberrant DNA secondary structures, further burden the replication process [47,63,64]. Critically, oncogene activation, notably of rat sarcoma viral oncogene homolog (RAS), Myelocytomatosis oncogene (MYC), and cyclin E, exacerbates RS through mechanisms such as increased replication origin firing, replication-transcription conflicts, and disrupted nucleotide metabolism. These oncogene-driven events induce pathological RS that often overwhelms the DDR system, particularly when key DDR components are dysfunctional. As a result, persistent RS leads to replication fork collapse, double-strand breaks (DSBs), and chromosomal rearrangements, all of which promote tumor heterogeneity and malignant progression. To counteract this, cells rely on a coordinated DDR network with a key role of ATR-CHK1 kinases [65].

ATR is a key serine/threonine kinase that plays a crucial role in DDR, particularly during RS and when single-stranded DNA (ssDNA) accumulates. When ssDNA forms, it is rapidly coated by replication protein A (RPA), composed of several subunits. In response to DNA damage, the RPA32 subunit undergoes phosphorylation at specific sites: Ser-23 and Ser-29, mediated by cyclin-dependent kinases 1/2 (CDK1 and CDK2), respectively. This phosphorylation is believed to depend on prior phosphorylation of Ser-33 by ATR [66]. RPA, in complex with ATR-interacting protein (ATRIP), facilitates ATR recruitment and activation; nevertheless, it is not the critical factor for kinase activation. CDK2 may also influence ATR activity by phosphorylating ATRIP at Ser-224 [67]. Full activation of ATR requires additional components, including DNA topoisomerase 2-binding protein 1 (TOPBP1) and the RAD9-RAD1-HUS1 (9-1-1) complex [68,69,70]. A recently identified ATR activator, Ewing’s tumor-associated antigen 1 (ETAA1), binds RPA and promotes ATR activation during DNA damage and replication fork restart [71,72]. Additionally, the TIMELESS/TIPIN complex strengthens the interaction between CLASPIN and RPA [73,74,75,76,77]. Early in the damage response, ATR also phosphorylates histone H2AX at Ser-319, resulting in the formation of γH2AX, a key marker of DNA damage [78].

**Figure 2:**
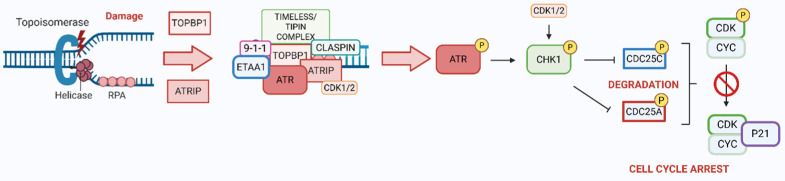
Ataxia telangiectasia and Rad3-related (ATR) kinase and checkpoint kinase 1 (CHK1) mediate cell cycle arrest in response to replication stress (RS) and DNA damage. During RS or single-stranded DNA (ssDNA) accumulation, replication protein A (RPA) coats ssDNA, enabling recruitment of ATR via ATR-interacting protein (ATRIP). ATR phosphorylates RPA32 at Ser-33, followed by cyclin-dependent kinases 1/2 (CDK1/2)-mediated phosphorylation at Ser-23 and Ser-29. ATR activation is further enhanced by topoisomerase 2-binding protein 1 (TOPBP1), the RAD9-RAD1-HUS1 (9-1-1) complex, and Ewing’s tumor-associated antigen 1 (ETAA1). The TIMELESS/TIPIN complex also supports CLASPIN-RPA interaction. Once active, ATR phosphorylates histone H2AX at Ser-319 (forming γH2AX) and CHK1 at Ser-317 and Ser-345. Additional CDK1/2 phosphorylation of CHK1 at Ser-286 and Ser-301 enhances checkpoint signaling. Activated CHK1 phosphorylates cell division cycle 25 (CDC25) phosphatases—CDC25A and CDC25C—leading to their degradation. This prevents activation of cyclin-dependent kinases (CDKs), halting G1/S and G2/M transitions and allowing DNA repair. Created in BioRender. Kciuk, M. (2025) https://BioRender.com/7vihaky (accessed on 8 August 2025).

Once activated, ATR phosphorylates several downstream effectors, most notably checkpoint kinase 1 (CHK1) at Ser-317 and Ser-345 residues [79,80,81]. Additionally, CHK1 can be phosphorylated at Ser-286 and Ser-301 by CDK1 and CDK2. This CDK-dependent phosphorylation becomes especially important after DNA damage or RS, as it facilitates full CHK1 activation and ensures proper checkpoint function [82]. CHK1 plays a regulatory role over two key M-phase inducer phosphatases, cell division cycle 25C and 25A (CDC25C, CDC25A). Under physiological conditions, CDC25 phosphatases activate CDK-cyclin complexes, promoting progression through the cell cycle. CDC25C primarily facilitates the G2/M transition by removing inhibitory phosphates from CDK1 at threonine 14 (T14) and tyrosine 15 (Y15). In contrast, CDC25A has a broader role, contributing to both G1/S and G2/M cell cycle progression. It achieves this by dephosphorylating CDK4 at Y17, CDK6 at Y24, and both CDK2 and CDK1 at the T14 and Y15 residues [83]. However, in the event of DNA damage, CHK1 becomes activated and phosphorylates CDC25 proteins, marking them for degradation. This prevents CDK1/2 activation, thereby halting cell cycle progression to allow time for DNA repair as presented in Figure 2 [83,84].

An alternative pathway for CHK1 activation has been identified, which becomes prominent when ATR is inhibited. In this context, the accumulation of ssDNA leads to the formation of abnormal DNA structures. These structures are processed by the SLX4-MUS81 endonuclease complex, composed of the SLX4 scaffold protein and the MUS81 structure-specific nuclease. This event activates CHK1 through a DNA-dependent protein kinase catalytic subunit (DNA-PKcs)-dependent mechanism, bypassing the conventional ATR-mediated activation route [85,86].

**Figure 3:**
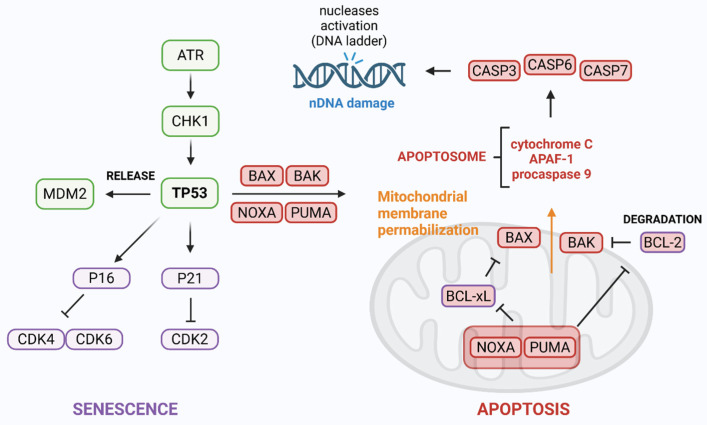
Ataxia telangiectasia and Rad3-related (ATR) kinase and checkpoint kinase 1 (CHK1) play key roles in apoptosis and senescence. Following DNA damage, ATR phosphorylates tumor protein p53 (TP53), leading to its release from E3 ubiquitin-protein ligase (MDM2), stabilization, and activation. Activated TP53 promotes transcription of cyclin-dependent kinase inhibitors CDKN1A (P21) and CDKN2A (P16), inducing G1/S cell cycle arrest. TP53 also upregulates pro-apoptotic proteins, including BCL2-associated X (BAX), BCL2 antagonist/killer 1 (BAK), phorbol-12-myristate-13-acetate-induced protein 1 (NOXA), and BCL2 binding component 3 (PUMA), promoting mitochondrial outer membrane permeabilization and cytochrome c release. This activates the apoptosome, triggering the caspase cascade. Executioner caspases 3, 6, and 7 activate caspase-activated DNase (CAD), resulting in DNA fragmentation characteristic of apoptosis. Created in BioRender. Kciuk, M. (2025) https://BioRender.com/ds10k1n (accessed on 8 August 2025).

ATR can also directly phosphorylate the tumor suppressor protein TP53 [87]. This modification is associated with the dissociation of its negative regulator, E3 ubiquitin-protein ligase (MDM2) [88]. TP53 is one of the most extensively studied tumor suppressors, with mutations in its gene found in approximately 50% of all human cancers. Following DNA damage, phosphorylated TP53 assembles into a tetrameric transcription factor that activates the expression of several target genes, including *CDKN1A* and *CDKN2A* (encoding the cyclin-dependent kinase inhibitors P21 and P16, respectively) or *BAX*, *BAK*, *NOXA,* and *PUMA* (for full names of the genes and proteins, see the abbreviation section), which are involved in promoting apoptosis. P21 and P16, upregulated by TP53, bind to and inhibit cyclin-CDK complexes, resulting in cell cycle arrest. It also prevents CDKs from phosphorylating the retinoblastoma protein (pRB), effectively blocking the transition from the G1 to the S phase of the cell cycle [89,90,91]. Altogether, these mechanisms contribute to apoptosis and senescence (Figure 3). This topic was previously reviewed by our group [92,93] and others [94,95,96].

Furthermore, ATR–CHK1 signaling axis plays a pivotal role in preserving replication integrity by tightly coordinating DNA replication origin firing. Under normal and stressed conditions, ATR-mediated checkpoint activation suppresses CDK and DBF4-dependent kinase (DDK) activity, thus limiting the unscheduled activation of replication origins. Mechanistically, the origin recognition complex (ORC) facilitates the loading of MCM2–7 helicase complexes onto DNA as inactive double hexamers. For replication initiation, these helicases require activation through CDK-dependent phosphorylation of Treslin and DDK-mediated phosphorylation of the MCM2–7 complex, promoting recruitment of cell division cycle 45 (CDC45). However, upon DNA damage or RS, ATR–CHK1 signaling inhibits CDK and DDK, thereby preventing CDC45 loading and helicase activation, effectively delaying replication origin firing to allow repair and fork stabilization. Additionally, ATR signaling stabilizes mixed-lineage leukemia protein (MLL) on chromatin, leading to histone H3K4 methylation at late replication origins, which further represses origin firing. Disruption of this finely tuned regulation, such as by pharmacological inhibition of ATR, results in uncontrolled origin activation and excessive ssDNA formation, overwhelming the RPA reservoir. This exhaustion of RPA compromises fork stability and culminates in replication fork collapse, DSBs, and ultimately replication catastrophe as reviewed by other authors [65,97,98].

## 3. ATR and CHK1 Kinases in Gastric Cancer

Genomic stability is critically dependent on an intact DDR and functional DNA repair mechanisms. In gastric cancers characterized by microsatellite instability (MSI), the loss of mismatch repair (MMR) function leads to an accumulation of insertion/deletion mutations [99]. In one of the earliest studies by Menoyo et al., the authors highlighted a high frequency of frameshift mutations in key genes of the DDR network, particularly the ATR–CHK1–CDC25C signaling axis and MMR components in gastric cancer. Concurrently, frequent mutations in MMR-associated genes such as MutS homolog 3 (*hMSH3*) (56%) and MutS homolog 6 (*hMSH6*) (43%), along with mediator complex subunit 1 (*MED1*) (43%), a base excision repair-associated gene, suggest an expansion of the mutator phenotype beyond canonical MMR defects. MED1, hMSH3, and hMSH6 may act as secondary mutators, promoting further genomic instability through defective post-replicative mismatch repair. The absence of mutations in other DDR-related genes such as ataxia-telangiectasia mutated (*ATM*), *BRCA1*, mutL homolog 3 (*hMLH3*), and Nijmegen breakage syndrome protein 1 (*NBS1*) suggested a selective vulnerability of the ATR-CHK1 axis in MSI-high gastric cancers. Collectively, these data underscored a functional impairment of the ATR-CHK1 pathway as a potential driver of gastric tumorigenesis in the MSI context, and proposed ATR and CHK1 as therapeutic vulnerabilities [100].

Falchetti et al. revealed in a population-based cohort of 159 gastric cancer patients that MSI-high tumors were significantly associated with specific clinicopathologic features, including antral tumor localization, intestinal histologic subtype, expanding growth pattern, and mucinous differentiation. This supported the notion that MSI-high gastric cancers arise via a unique tumorigenic pathway. Importantly, MSI-high status conferred a substantial survival benefit, with significantly higher 15-year survival rates, aligning with prior evidence that these tumors may elicit more robust immune responses due to high neoantigen load. Immunohistochemical loss of mutL homolog 1 (hMLH1) further correlated with expanding-type tumors, suggesting a mechanistic link between MMR deficiency and altered tumor invasion dynamics. On the molecular level, MSI-high gastric cancers exhibited a multigenic mutational spectrum, accumulating frameshift mutations across several critical pathways, including cell growth regulation (e.g., *TGFβRII*, *IGFIIR*, *TCF4*), apoptosis (e.g., *BAX*, *FAS*, *CASP5*), and DNA repair (e.g., *hMSH3*, *MED1*, *ATR*, *BRCA2*, *RAD50*) (for full names of the genes and proteins, see the abbreviation section). These mutations reflect the pervasive impact of replication errors in short coding repeats in the context of MMR deficiency. Furthermore, *RAD50* mutations were associated with significantly worse survival, highlighting a subset of MSI-high tumors with aggressive clinical behavior, potentially due to impaired double-strand break repair (DSBR) pathways [101].

Homologous recombination (HR) is a critical DNA repair mechanism that maintains genomic stability by accurately resolving DSBs. Dysregulation of key HR pathway proteins may contribute to gastric carcinogenesis by increasing genomic instability and promoting tumor progression. In an immunohistochemical study of 120 treatment-naïve gastric cancer samples, the expression loss of central HR-related proteins, BRCA1, ATM, ATR, mediator of DNA damage checkpoint 1 (MDC1), and meiotic recombination 11 homolog A (MRE11), was investigated in relation to clinicopathologic parameters. The most frequently deficient markers were BRCA1 (21.4%) and ATR (21.0%), followed by ATM (20.2%), MDC1 (11.1%), and MRE11 (4.6%). Notably, BRCA1 loss was significantly associated with aggressive disease features, including the diffuse histological subtype, high tumor grade, and advanced clinical stage. Similarly, MDC1 deficiency correlated with diffuse subtype and higher-grade tumors, indicating that its loss may also contribute to aggressive gastric cancer phenotypes. Furthermore, the loss of BRCA1 expression was a negative prognostic indicator, with a significantly reduced 2-year survival rate (32.4%) compared to BRCA1-positive tumors (62.8%). Although ATM, ATR, and MRE11 loss did not show significant clinicopathologic associations in this cohort, their known biological roles in DDR warranted further exploration in larger or more molecularly stratified datasets. These findings supported the hypothesis that homologous recombination deficiency (HRD), particularly through loss of BRCA1 and MDC1, is involved in the pathogenesis and progression of gastric cancer [102].

Among 51 individuals with gastric cancer, pathogenic or likely pathogenic variants were detected in 35% (18/51) cases, with a notable enrichment in genes associated with DDR, including *ATM*, *ATR*, *BRCA2*, BRCA1 interacting protein C-terminal helicase 1 (*BRIP1*), Fanconi Anemia complementation group C (*FANCC*), and *TP53*. Importantly, individuals harboring these variants were diagnosed at a younger age and more frequently reported a family history of gastric cancer, suggesting that early onset and familial clustering are useful clinical indicators for genetic testing. The detection of alterations in clinically actionable genes such as *ATM*, *ATR*, and *BRCA2* opens potential avenues for risk-reducing strategies and tailored therapeutic interventions, including PARP or ATR inhibitors (ATRis) [103].

In contrast, the study of Fewings et al. addressed a critical gap in understanding the genetic basis of hereditary diffuse gastric cancer (HDGC) in families lacking pathogenic cadherin 1 (*CDH1*) variants. Through whole-exome sequencing of 39 individuals across 22 such families, the researchers identified germline loss-of-function variants in several genes implicated in cancer predisposition and DNA repair pathways. Notably, a frameshift deletion in the partner and localizer of BRCA2 (*PALB2*), a well-established breast cancer susceptibility gene involved in HR-mediated DSBs repair, was found in a family with a history of both gastric and breast cancers, suggesting *PALB2* as a novel contributor to HDGC risk. Additionally, variants in the mismatch repair gene *MSH2*, including a frameshift insertion and a start-codon loss, implicate defects in DNA mismatch repair mechanisms in a subset of these families. Moreover, the detection of variants in *ATR*, *NBS*, and RecQ-like helicase 5 (*RECQL5*) points to broader involvement of DDR dysfunction in HDGC predisposition beyond the classical *CDH1* pathway. The identification of *RECQL5* variants in two unrelated families is particularly intriguing, indicating potential new candidate genes that warrant further functional and epidemiological validation. This study, therefore, expands the genetic landscape of hereditary diffuse gastric cancer and emphasizes the heterogeneity of its molecular etiology. Clinically, these findings advocate for the inclusion of *PALB2*, mismatch repair, and other DNA repair genes in genetic testing panels for families with HDGC but negative for *CDH1* mutations, potentially improving risk stratification and informing surveillance or preventive strategies. The results also raise the possibility of overlapping cancer susceptibilities, highlighting the importance of personalized risk assessment in hereditary cancer syndromes. Further research to confirm these associations and understand the mechanisms by which these genes contribute to gastric tumorigenesis will be vital for developing targeted interventions in this high-risk population [104].

Another study explored the prognostic significance of RAD51, BRCA2, ATM, ATR, BRCA1, CHK2, γH2AX, and TP53 expression in gastric adenocarcinoma. Analyzing 121 advanced gastric cancer surgical specimens, the authors found that loss of nuclear RADiation sensitive 51 (RAD51) expression was associated with more aggressive tumor features such as vascular invasion, lymph node metastasis, and larger tumor size. Importantly, RAD51 negativity correlated with poorer overall and disease-free survival in univariate analysis, highlighting its potential as a negative prognostic biomarker. Interestingly, despite this, RAD51-negative tumors showed a better response to adjuvant therapy, suggesting RAD51 status may also predict treatment sensitivity. Nuclear ATR negativity was linked with larger tumors and higher histological grade, consistent with a role in tumor progression. Conversely, ATM positivity was associated with improved disease-free survival, supporting its function as a tumor suppressor via DNA repair. The expression of nuclear BRCA2 showed complex correlations: while its positivity was associated with lower overall survival and more aggressive diffuse histological type, higher BRCA2 levels also correlated with vascular invasion. Paradoxically, BRCA2-positive tumors were more often low grade and intestinal-type, indicating heterogeneity in BRCA2’s prognostic implications depending on tumor subtype. Collectively, these findings underscore the importance of HR proteins as prognostic biomarkers in gastric adenocarcinoma. RAD51 and BRCA2, in particular, show promise for predicting tumor aggressiveness and patient outcomes, as well as response to therapy. This knowledge could inform personalized treatment strategies by integrating HR protein expression profiles into clinical decision-making. Further studies are warranted to validate these markers prospectively and to elucidate their mechanistic roles in gastric cancer progression and therapy response [105].

Zeng et al. addressed the clinical utility of next-generation sequencing (NGS) in gastric cancer by developing a custom 24-gene panel designed to detect common somatic mutations and copy number variations relevant to gastric cancer diagnosis and targeted therapy. By focusing on high-frequency cancer driver and tumor suppressor genes, the panel aimed to facilitate personalized treatment decisions. The results highlighted several frequently mutated genes in gastric cancer, including *TP53* (58%), erb-b2 receptor tyrosine kinase 2 (*ERBB2; HER2*) (28%), and *BRCA2* (23%), confirming their known roles in gastric tumorigenesis. Other notable mutated genes included *ATR* (14%), phosphatidylinositol-4,5-bisphosphate 3-kinase catalytic subunit alpha (*PIK3CA*) (14%), *ATM* (9%), and *MSH2* (12%). The mutation frequencies largely matched those reported in the large cBioPortal cancer genomics database, supporting the panel’s relevance. Importantly, enrichment analysis revealed that many of the frequently mutated genes are involved in transmembrane receptor protein kinase activity, which is critical for cancer cell growth and survival and serves as a target for various therapies. The identification of mutations in *BRCA2*, *PIK3CA*, and fibroblast growth factor receptor 2 (*FGFR2*) genes particularly underscored their potential as predictive biomarkers and therapeutic targets in gastric cancer. Clinically, the study included both MSS and MSI high cases, which is important given the impact of MSI status on immunotherapy responsiveness. The integration of mutation data with databases such as ClinVar and OncoKB further aids in linking specific mutations to actionable treatments [106].

The results highlight that epigenetic mechanisms, particularly methylation of *RAD51*, *BRCA1*, and *BRCA2* genes, are frequently involved in gastric cancer development and progression. This methylation likely leads to reduced expression of these critical HR repair proteins, supporting the hypothesis that decreased *BRCA1* expression contributes to disease progression. Reduced DNA repair capacity can promote genomic instability, fueling cancer development and aggressiveness. Interestingly, the study reports a significant association between *RAD51* mRNA levels and perineural invasion as well as patient mortality. This finding contrasts with earlier immunohistochemical studies that focused on RAD51 protein levels, suggesting a complex regulation of RAD51 where mRNA abundance does not necessarily correlate with protein expression or function. Such discrepancies point toward post-transcriptional or post-translational regulation mechanisms impacting RAD51 activity in gastric cancer. Moreover, a similar lack of correlation, and in some cases inverse correlation, between *ATR* mRNA and protein expression further supports the idea that regulation beyond gene transcription is at play. This may include mRNA stability, translation efficiency, protein modification, or degradation pathways, which could modulate the functional levels of ATR independently of its transcript abundance. These findings emphasize the importance of integrating multiple molecular layers (epigenetics, transcriptomics, proteomics) to fully understand DNA repair dysregulation in gastric cancer. They also suggest that relying solely on mRNA or protein measurements might provide incomplete or misleading insights, highlighting the need for further investigation into post-transcriptional and post-translational regulatory mechanisms influencing key DNA repair factors like RAD51 and ATR [107].

## 4. ATR Kinase Inhibitors in Gastric Cancer

### 4.1. Pre-Clinical Studies

In one of the first studies, researchers tested VE-821, as a specific ATR inhibitor, to explore its impact on cisplatin’s effectiveness in two gastric cancer cell lines (MKN-45 and AGS). Cisplatin alone inhibited proliferation and induced apoptosis, but at relatively high concentrations, reflecting the limited sensitivity of cells to this therapeutic agent. Cisplatin treatment also increased phosphorylation of ATR, CHK1, and γ-H2AX, indicating activation of the DDR pathway. VE-821 similarly inhibited cell proliferation and induced apoptosis, but by blocking phosphorylation of ATR and CHK1, and as a result, effectively suppressed the DDR. Importantly, VE-821 reversed cisplatin-induced activation of signal transducer and activator of transcription 3 (STAT3), a signaling molecule often involved in cancer survival, and further increased γ-H2AX levels, indicating enhanced DNA damage. Combining VE-821 with cisplatin resulted in synergistic effects, significantly increasing the sensitivity of gastric cancer cells and cell organoids to cisplatin treatment. This synergy was also observed in vivo, suggesting potential clinical relevance. Analysis of the Cancer Genome Atlas (TCGA) database revealed that higher ATR expression correlated with more advanced pathological stages in gastric cancer patients, reinforcing the clinical significance of ATR in disease progression and therapy resistance. In conclusion, this study demonstrated that VE-821 sensitizes gastric cancer cells to cisplatin by inhibiting the ATR-mediated DDR pathway and reversing cisplatin-induced survival signaling [108].

Another study indicated p21 (RAC1) activated kinase 6 (PAK6) as a critical regulator of the DDR and chemoresistance in gastric cancer, particularly in relation to oxaliplatin, another commonly used bifunctional alkylating agent. Clinical analysis showed that higher *PAK6* expression correlates with more advanced tumor stages, deeper invasion, increased lymph node metastasis, higher rates of recurrence, and notably, resistance to oxaliplatin treatment. This indicates that *PAK6* expression may serve as a biomarker for aggressive disease and poor chemotherapy response. Functionally, PAK6 promotes chemoresistance by enhancing HR. It translocates into the nucleus, where it activates ATR kinase. Activated ATR subsequently triggers CHK1 phosphorylation/activation and recruits RAD51 to DNA damage sites, facilitating efficient repair. This cascade protects gastric cancer cells from oxaliplatin-induced apoptosis, allowing tumor cells to survive despite chemotherapy. Importantly, the study demonstrated that inhibiting ATR using the specific inhibitor ceralasertib (AZD6738) can block PAK6-mediated HR repair. This blockage reversed oxaliplatin resistance and increased sensitivity to the drug, even in cells with high *PAK6* levels. This suggests that targeting ATR could be a promising therapeutic strategy to overcome chemoresistance in patients whose tumors overexpress *PAK6*. In summary, PAK6 emerges as a novel modulator of DDR and chemoresistance in gastric cancer through its activation of the ATR-CHK1-RAD51 repair pathway. These findings not only deepen our understanding of the molecular mechanisms driving chemotherapy resistance but also propose ATR inhibition as a viable approach to improve treatment efficacy in PAK6-high gastric cancers [109].

The study by Min et al. explored the efficacy of ATR inhibition using AZD6738 in gastric cancer models with compromised ATM function. In *ATM*-deficient SNU-601 cells, treatment with AZD6738 resulted in marked accumulation of DNA damage, evidenced by the reduced RAD51 foci formation, S-phase cell cycle arrest, and increased caspase 3-mediated apoptosis. In contrast, SNU-484 cells with intact ATM signaling were resistant to AZD6738 monotherapy. However, pharmacological inhibition of ATM in SNU-484 cells restored sensitivity to AZD6738, underscoring the synthetic lethality between ATR inhibition and ATM deficiency. Mechanistically, the study also uncovered that reduced histone deacetylase 1 (HDAC1) expression in *ATM*-deficient cells might further influence AZD6738 sensitivity, suggesting crosstalk between chromatin remodeling and DDR pathways. Importantly, in vivo xenograft models confirmed the antitumor efficacy of AZD6738, demonstrating significant tumor growth inhibition and increased apoptosis in *ATM*-deficient tumors. These findings strongly support the concept of synthetic lethality between ATR inhibition and *ATM* deficiency in GC, offering a rationale for biomarker-driven clinical trials. Specifically, selecting patients with *ATM* loss or low *HDAC1* expression could optimize responses to ATR-targeted therapies such as AZD6738 [110].

#### Completed Clinical Trials

A phase II clinical trial (NCT03780608) investigated the therapeutic potential of combining AZD6738 with durvalumab, a programmed death-ligand 1 (PD-L1) immune checkpoint inhibitor, in patients with previously treated advanced gastric cancer. The rationale for this combination stems from emerging evidence that inhibition of the DDR, particularly through ATR blockade, not only enhances cancer cell death but also amplifies anti-tumor immune responses. ATR inhibition leads to the accumulation of cytosolic DNA fragments that activate the cyclic GMP-AMP synthase-stimulator of interferon genes (cGAS-STING) pathway, a key sensor of DNA damage that stimulates type I interferon signaling and promotes immune-mediated tumor clearance. By disrupting DNA repair, ceralasertib may potentiate the immunogenicity of tumors, thereby enhancing the efficacy of immune checkpoint inhibitors such as durvalumab. The study demonstrated a modest overall response rate (ORR) of 22.6% and a disease control rate (DCR) of 58.1%, with manageable toxicity, suggesting clinical activity of the combination in a heavily pretreated population. Importantly, biomarker analysis revealed that patients with *ATM* loss or an HR deficiency mutational signature experienced significantly improved progression-free survival (PFS), underscoring the relevance of DDR alterations as predictive biomarkers for ATR inhibitor sensitivity. Furthermore, immune profiling identified immune activation in responders, including increased intratumoral lymphocytes and expansion of tumor-reactive CD8^+^ T cells, providing mechanistic support for the observed clinical benefit. Conversely, resistance was associated with enrichment of a tumor vasculature signature, highlighting potential avenues for combinatorial strategies. These findings collectively support the hypothesis that targeting DDR pathways can sensitize immunologically “cold” tumors to checkpoint blockade, and provide a strong rationale for biomarker-driven trials of DDR-immune checkpoint inhibitor combinations in gastric cancer and other malignancies characterized by DDR defects [111]. Table 1 summarizes preclinical and clinical studies involving the use of ATRis in gastric cancer.

### 4.2. Ongoing and Future Clinical Studies

There are several clinical trials investigating ATRis in gastric cancer treatment registered on https://clinicaltrials.gov/ (accessed on 6 July 2025).

A modular phase I/1b, open-label, multicenter study (NCT02264678) was designed to explore the safety, tolerability, pharmacokinetics, and preliminary efficacy of AZD6738 in combination with a range of cytotoxic chemotherapies and novel anti-cancer agents in patients with advanced or metastatic solid tumors, including gastric cancer. By impairing ATR function, AZD6738 disrupts the repair of replication-associated DNA lesions, thereby sensitizing tumor cells to DNA-damaging agents such as platinum compounds and PARP inhibitors. The modular design of the trial will enable a flexible and adaptive framework to evaluate multiple drug combinations, beginning with carboplatin (Module 1), olaparib (Module 2), durvalumab (Module 3), and AZD5305, a next-generation PARP1-selective inhibitor (Module 5). The inclusion of both dose-escalation (Part A) and cohort expansion (Part B) components will allow for a thorough assessment of dose-limiting toxicities and preliminary signals of efficacy in biomarker-defined subgroups. Importantly, the study will include a dedicated module to assess the impact of food intake on drug absorption and potential effects on cardiac electrophysiology (QT interval), reflecting a comprehensive evaluation of safety and pharmacokinetics. The combination of AZD6738 with carboplatin or olaparib is supported by preclinical evidence of synthetic lethality, particularly in tumors with deficiencies in the HR pathway. Meanwhile, its combination with durvalumab aims to exploit the immunomodulatory effects of ATR inhibition, such as enhanced tumor immunogenicity and increased T-cell infiltration, as previously shown in DDR-deficient tumors. The inclusion of AZD5305 reflects ongoing efforts to refine DDR-targeted therapies with improved selectivity and safety profiles. Overall, this study represents a strategically adaptive platform to define optimal combination regimens of ATR inhibition and establish biologically active doses across diverse therapeutic contexts, ultimately informing subsequent biomarker-guided trials in molecularly defined cancer populations.

This second phase I/Ib clinical trial (NCT04704661) will explore the safety, pharmacodynamics, and preliminary efficacy of combining AZD6738 with trastuzumab deruxtecan (DS-8201a), an antibody–drug conjugate targeting HER2, in patients with advanced solid tumors harboring *HER2* gene or protein alterations. The scientific rationale for this combination lies in the complementary mechanisms of action between the two agents: trastuzumab deruxtecan selectively delivers a cytotoxic topoisomerase I inhibitor to *HER2*-expressing tumor cells, leading to DSBs, while AZD6738 inhibits the ATR-mediated DDR, thereby impairing the tumor’s ability to repair RS and DNA lesions. This dual assault on DNA integrity is hypothesized to potentiate tumor cell death, particularly in tumors with underlying DNA repair deficiencies or RS. The trial includes a dose-escalation phase to establish the recommended phase II dose and evaluate tolerability, followed by a dose-expansion phase comparing the combination to monotherapy in colorectal and gastroesophageal cancers with *HER2* expression. Importantly, the study incorporates an innovative pharmacodynamic component, examining DNA damage markers (e.g., phosphorylated RAD50, schlafen family member 11 (SLFN11)) in tumor biopsies, and compares the effects of topoisomerase I inhibition alone compared to dual topoisomerase and ATR inhibition on DNA repair pathways. The design further allows for comprehensive exploration of predictive biomarkers, such as HER2 heterogeneity, *TP53*, *ATM*, and *RAS* alterations, which may inform patient selection strategies for future studies. Preliminary pharmacokinetic and immunogenicity analyses will also be performed, along with the development of a biorepository of tissue, blood, and preclinical models for ongoing translational research. Notably, a run-in period of trastuzumab deruxtecan monotherapy in the first cycle of the expansion phase will allow for within-patient comparison of molecular changes before and after AZD6738 exposure.

Gastric and gastroesophageal junction (GEJ) cancers represent a significant global health burden, with advanced-stage disease often associated with poor prognosis and limited treatment options. Standard second-line chemotherapy, including irinotecan monotherapy, demonstrates modest efficacy, with historical ORR of approximately 5%. Recent insights into the molecular underpinnings of gastric/GEJ cancer have highlighted frequent alterations in DDR pathways, particularly mutations in *TP53*, which occur in a substantial subset of patients. *TP53* mutations abrogate the G1/S cell cycle checkpoint, thereby increasing tumor cell reliance on the ATR-mediated G2/M checkpoint for DNA damage repair and survival. This dependency creates a therapeutic vulnerability that can be exploited through synthetic lethality using berzosertib (M6620) first-in-class ATR inhibitor. Phase II clinical trial (NCT03641313) will evaluate the efficacy of berzosertib in combination with irinotecan in patients with progressive, metastatic, or unresectable gastric/GEJ cancer, with a specific focus on *TP53*-mutant tumors. The primary objective is to assess improvement in ORR compared to historical controls, with secondary endpoints including PFS, OS, duration of response (DOR), and time to progression (TTP). Correlative biomarker analyses are incorporated to confirm target engagement and explore predictive markers of response, including additional DDR gene alterations. This study aims to establish a biomarker-driven therapeutic strategy that may significantly enhance outcomes in a molecularly defined subset of patients with advanced gastric and GEJ cancers.

Two other clinical trials will investigate elimusertib (BAY 1895344), another promising ATR inhibitor in gastric cancer. The phase I study (NCT04535401) will investigate the safety, tolerability, and preliminary efficacy of elimusertib in combination with FOLFIRI (leucovorin calcium, fluorouracil, and irinotecan hydrochloride), a standard regimen in gastrointestinal malignancies. The primary objective is to determine the maximum tolerated dose (MTD) of elimusertib when administered with FOLFIRI. Secondary endpoints include ORR, PFS, OS, and clinical benefit rate, while pharmacokinetics and DNA damage biomarker analyses in tumor tissue and peripheral blood mononuclear cells (PBMCs) will further elucidate the mechanistic effects of the combination. Exploratory aims assess the influence of uridine 5′-diphospho-glucuronosyltransferase family 1 member A1 (UGT1A1), tumor mutation profiles, and *ATM* expression on treatment response and toxicity. The trial follows a dose-escalation design, followed by expansion in selected cohorts, and integrates paired biopsies and serial imaging to evaluate on-treatment biological changes. This study aims to establish a biologically informed combination regimen that capitalizes on ATM pathway inhibition to sensitize gastrointestinal tumors to chemotherapy-induced DNA damage.

Another phase I clinical trial (NCT04491942) will evaluate the safety, tolerability, and optimal dosing of elimusertib in combination with cisplatin alone or with cisplatin plus gemcitabine in patients with advanced solid tumors, including those with metastatic gastric cancer. The trial follows a dose-escalation design with two treatment arms: a doublet (cisplatin + elimusertib) and a triplet regimen (cisplatin + gemcitabine + elimusertib). The primary objectives are to establish the MTD, recommended phase II dose, and the overall safety profile of each combination. Secondary endpoints include the pharmacokinetic characterization of BAY 1895344 and gemcitabine, and preliminary assessment of anti-tumor activity. Exploratory analyses investigate correlations between clinical outcomes and biomarkers of DDR deficiency, including somatic gene alterations and circulating tumor DNA (ctDNA), using comprehensive genomic and transcriptomic profiling. By integrating molecular biomarker analysis with conventional clinical endpoints, this study aims to lay the foundation for a biomarker-informed strategy that enhances the efficacy of chemotherapy through selective ATR inhibition in molecularly defined subsets of patients with advanced solid tumors.

Ongoing and future clinical studies investigating ATRis are provided in Table 2.

## 5. CHK1 Inhibitors in Gastric Cancer

While *CHK1* is upregulated in a subset of gastric carcinomas, its expression does not show a statistically significant association with *TP53* mutations, unlike *CHK2*. However, its correlation with TP53 protein levels suggests a potential indirect regulatory relationship, possibly reflecting checkpoint activation in response to genomic instability. CHK1 may still play a functional role in cell cycle arrest in the context of TP53 dysfunction; however exact mechanistic contribution is still not clear [112].

In the comprehensive analysis of 136 MSI-high carcinomas originating from gastric, colorectal, and endometrial tissues, *CHK1* was investigated as one of seven target genes frequently affected by frameshift mutations in coding region microsatellites (CDRs). The study employed immunohistochemical detection of MMR proteins hMLH1 and hMSH2, along with microsatellite analysis, to correlate the mutational status of *CHK1* and other target genes with clinico-pathological parameters and overall instability burden. The findings support a model wherein the MMR gene inactivation, primarily through *hMLH1* loss, triggers a cascade of mutations in downstream genes, including *CHK1*, contributing to MSI-high tumorigenesis. While mutations in *TGFβRII* and *BAX* appeared early in tumor development, *CHK1* frameshifts occurred at later stages, suggesting a potential role in the progression rather than initiation of MSI-high neoplasms. Interestingly, gastrointestinal tumors harboring early Bat-26 and Bat-25 alterations demonstrated a more indolent clinical course and were often diagnosed at earlier stages, typically showing *TGFβRII* and *BAX* mutations prior to CHK1 involvement. Conversely, advanced-stage MSI-high tumors showed a heterogeneous mutational landscape, with some displaying a ‘mild mutator phenotype’ and few CDR frameshifts, including in *CHK1*. Notably, a lower burden of CDR frameshifts, including those affecting CHK1, was significantly associated with lymph node metastasis, suggesting that in a subset of tumors, aggressive biological behavior may arise through mechanisms beyond the classical mutator phenotype. These results imply that *CHK1* mutation, while a marker of genomic instability in MSI-high tumors, may also serve as an indicator of divergent pathways of tumor progression, potentially integrating both mutator and suppressor mechanisms [113].

In an effort to elucidate molecular contributors to gastric cancer biology, a study by Arai et al. employed comprehensive protein profiling via liquid chromatography-tandem mass spectrometry on formalin-fixed paraffin-embedded samples from 17 gastric cancer patients. Initial analyses identified multiple DDR-related proteins, including CHK1, CHK2, X-ray repair cross-complementing 6 (KU70), and interleukin enhancer binding factor 2 (ILF2), as potential candidates. To further assess their functional status, the expression and phosphorylation of CHK1, CHK2, KU70, and ILF2 were examined by immunohistochemistry in an expanded cohort of 42 gastric cancer cases. While CHK1 was consistently expressed across all samples, its phosphorylation status, critical for activation, was not explicitly impaired, distinguishing it from CHK2, which exhibited absent phosphorylation in a subset of tumors. These findings suggest that CHK1 is constitutively expressed but may not always be functionally active, and that defects in the broader DDR pathway, rather than CHK1 specifically, may underlie impaired DNA repair mechanisms in a subset of gastric cancers. Supporting this, 17% (7/42) of cases demonstrated impaired DDR protein activation, which was associated with a higher recurrence rate (29%) compared to tumors with intact DDR (6%). In vitro analysis using four gastric cancer cell lines further demonstrated that only one line exhibited a complete DDR response, including sequential phosphorylation of CHK1 and CHK2, TP53 upregulation, and apoptosis, upon UV-induced DNA damage. The remaining lines showed abnormal CHK1 and CHK2 phosphorylation dynamics, implicating dysfunctional DDR as a potential determinant of therapeutic resistance and tumor aggressiveness. These findings position CHK1 as a constitutively present yet contextually regulated component of the DDR machinery in gastric cancer, and highlight the necessity of its proper activation, alongside TP53 and CHK2, for effective cellular response to genotoxic stress [114].

Given its involvement in the cellular response to genotoxic stress, a study by Bargiela-Iparraguirre et al. evaluated CHK1 expression in AGS and MKN45 gastric cell lines and a small cohort of patient tumor samples. The findings revealed that overexpression of CHK1 significantly enhanced resistance to radiation, suggesting a protective role against DNA-damaging therapies. Functional inhibition of CHK1, via the pharmacological inhibitor UCN-01 or gene silencing with short hairpin RNA (shRNA) targeting CHK1, sensitized gastric cancer cells to radiation and bleomycin, further supporting its role as a modulator of therapeutic resistance. Clinically, nuclear accumulation of CHK1 protein in patient tumors was associated with decreased PFS, highlighting its potential prognostic value. Mechanistically, CHK1 expression was shown to be under transcriptional regulation by the TP53 and RB/E2F pathways. Additionally, preliminary data identified a post-transcriptional regulatory layer involving miR-195 and miR-503, whose downregulation in radioresistant cells correlated inversely with elevated CHK1 levels. Together, these results implicate a CHK1/miRNA regulatory axis in radiation resistance in gastric cancer and support the utility of CHK1 as a predictive biomarker for radiotherapy response, potentially aiding in the stratification of patients for adjuvant treatment following surgery [115].

The therapeutic utility of PARP inhibitors in cancers with intact BRCA1/2 function has traditionally been limited due to the retention of HR repair capacity. However, recent strategies have explored disrupting HR by targeting key regulators, including CHK1, particularly in the context of TP53-deficient tumors, which often rely heavily on CHK1-mediated checkpoints for DNA damage tolerance and survival [116].

Study by Yin et al. highlighted CHK1 as a critical regulator of gastric cancer cell survival and proliferation, underscoring its potential as a therapeutic target. Functional ablation of CHK1 via siRNA markedly inhibited proliferation and enhanced radiosensitivity in both TP53 wild-type (AGS) and TP53-mutant (MKN1) gastric cancer cell lines, indicating that CHK1-driven tumor growth and therapeutic resistance may be independent of TP53 status. Additionally, the use of LY2606368 (prexasertib), as a selective CHK1/CHK2 inhibitor, demonstrated potent anti-cancer activity. LY2606368 induced substantial DNA damage, inhibited proliferation, and triggered apoptosis in both cell lines. Notably, the compound also suppressed HR-mediated DNA repair, suggesting that CHK1 plays a central role in maintaining genomic integrity in gastric cancer cells. These effects were further amplified when LY2606368 was combined with BMN673 (talazoparib), a PARP1 inhibitor, resulting in synergistic anti-tumor activity in both in vitro models and patient-derived xenograft (PDX) systems. Mechanistically, the synergy was attributed to the disruption of the G2/M checkpoint by CHK1 inhibition, which forced damaged cells into premature mitosis and subsequent cell death in the presence of unrepaired DNA. Collectively, the study provides compelling evidence that CHK1-targeted therapies, especially in combination with PARP inhibitors, may represent an effective strategy for the treatment of gastric cancer, irrespective of TP53 mutational status [117].

A study by Zhao et al. investigated the chemopotentiating effect of CHK1 inhibition using MK-8776 in BRCA-proficient, TP53-null cancer cell lines (AsPC-1 and H1299). These cells were initially resistant to olaparib, a PARP inhibitor, unlike BRCA1-mutant MDA-MB-436 cells. Co-treatment with MK-8776 and olaparib at doses lower than those contributing to the reduction in cell viability by half (IC_50_) induced a synergistic cytotoxic response, suppressing cell proliferation and enhancing apoptosis. Mechanistically, CHK1 inhibition impaired HR by disrupting olaparib-induced BRCA1 foci formation and replication fork stability, as evidenced by the loss of MCM7 loading and increased accumulation of γH2AX, a marker of DNA double-strand breaks. These findings illustrate how CHK1 inhibition effectively dismantles compensatory repair mechanisms in HR-proficient cells, converting a typically olaparib-insensitive phenotype into a synthetically lethal state. Moreover, even with the introduction of mutant TP53 (R175H), a known driver of therapy resistance, the combination retained its synergistic potency, albeit at lower concentration thresholds, suggesting that CHK1 inhibition may overcome TP53-associated resistance mechanisms. This study provides compelling rationale for the use of CHK1 inhibitors to expand the efficacy of PARPi therapies beyond BRCA-deficient settings, particularly in tumors where TP53 loss creates a dependence on CHK1 for genome integrity and cell cycle progression. These findings underscore a promising strategy to broaden PARP inhibitors applicability in a wider spectrum of solid tumors through rational, mechanism-based combination approaches targeting synthetic vulnerabilities [118].

Oncogene amplification, particularly when driven by extrachromosomal DNA (ecDNA), is a hallmark of aggressive solid tumors associated with poor prognosis, resistance to therapy, and intratumoral heterogeneity. These amplified oncogenes often induce heightened RS, creating a dependency on the DDR machinery, specifically the ATR-CHK1 pathway, for cell cycle regulation and survival. Inhibition of CHK1 therefore represents a rational strategy to exploit synthetic lethality in tumors harboring ecDNA-mediated oncogene amplification, selectively targeting tumor cells while sparing normal tissue. BBI-355 is a potent, selective, orally bioavailable CHK1 inhibitor under clinical investigation as an ecDNA-directed therapy (ecDTx). A first-in-human, open-label, multicenter Phase 1/2 trial (NCT05827614) has been designed to evaluate the safety, pharmacokinetics, and preliminary efficacy of BBI-355 as monotherapy and in combination with targeted therapies. This three-part, non-randomized, sequential assignment study includes dose-escalation and expansion cohorts across multiple arms: single-agent BBI-355, BBI-355 plus the epidermal growth factor receptor (EGFR) inhibitor erlotinib (in patients with EGFR-amplified tumors), and BBI-355 plus the FGFR inhibitor futibatinib (in patients with FGFR1/4 amplification). Eligible patients are adults with locally advanced or metastatic non-resectable solid tumors harboring oncogene amplifications, whose disease has progressed following standard therapies or for whom no alternative therapy exists. The study’s primary objectives are to determine the maximal tolerated dose and recommended phase II dose of BBI-355, both alone and in combination, and to characterize the treatment-emergent adverse event (TEAE). Secondary endpoints include pharmacokinetic parameters and anti-tumor activity. Exploratory analyses will evaluate molecular predictors of response, including amplification-specific dependencies, to inform future biomarker-driven development. By targeting the RS vulnerability in ecDNA-positive cancers, BBI-355 offers a novel therapeutic avenue aimed at overcoming genomic complexity and therapeutic resistance in molecularly defined tumor subsets.

## 6. Conclusions and Prospects

Gastric cancer frequently harbors molecular features that render it particularly susceptible to disruption of DDR, through inhibition of the ATR–CHK1 axis. High rates of *TP53* mutations, present in up to 50% of gastric tumors, contribute to defective G1 checkpoint control, leading to reliance on ATR–ATR-CHK1-mediated S and G2/M checkpoints for survival under RS. Oncogene activation, such as *MYC* amplification, and exposure to endogenous or therapeutic genotoxic stress further exacerbate RS in gastric tumor cells, elevating their dependency on ATR–CHK1 signaling for replication fork stabilization and cell cycle progression. In this context, ATR or CHK1 inhibition drives premature mitotic entry, causing mitotic catastrophe and TP53-independent cell death [119,120,121,122,123].

Moreover, ATR–CHK1 blockade induces the accumulation of cytosolic double-stranded DNA and micronuclei, which in turn activate the cGAS/STING pathway, leading to type I interferon responses and a pro-inflammatory tumor microenvironment. These effects are particularly relevant in gastric cancer, where immune checkpoint blockade has shown limited efficacy, in part due to an immunosuppressive milieu. ATR–CHK1 inhibition may prime tumors for immune attack by increasing tumor neoantigen burden, promoting MHC-I expression, and enhancing CD8^+^ T-cell infiltration, especially when combined with radiotherapy or immune checkpoint inhibitors. Preclinical evidence suggests that the use of ATRis such as AZD6738 or CHK1 inhibitors like AZD7762 can synergize with radiation or PD-1 blockade to stimulate anti-tumor immunity and delay tumor progression. Thus, targeting ATR–CHK1 not only exploits intrinsic vulnerabilities in gastric cancer cells but also transforms the tumor-immune interface, offering a promising strategy for overcoming therapeutic resistance and immunotherapy insensitivity in gastric cancer [119,124,125].

Early-phase clinical trials investigating ATRis have largely focused on their use in combination with chemotherapeutic agents that exacerbate RS, thereby potentiating ATRi efficacy. Chemotherapeutics such as antimetabolites (e.g., gemcitabine) impair DNA synthesis by both depleting nucleotide pools and incorporating nucleotide analogs that cause chain termination, while DNA crosslinking agents (e.g., cisplatin, carboplatin) and topoisomerase inhibitors (e.g., topotecan, irinotecan) create physical impediments to replication fork progression [126]. These agents heighten RS, making cancer cells increasingly reliant on ATR-mediated RS response pathways for survival. Preclinical studies employing ATRis such as VE-821 and ceralasertib (AZD6738) have demonstrated synergistic cytotoxic effects when combined with DNA-damaging therapies, particularly in cells harboring *ATM* deficiency and *TP53* loss, genetic backgrounds known to compromise DNA damage checkpoint fidelity. Notably, this combinatorial strategy selectively enhanced tumor cell death while sparing normal cells, underscoring a favorable therapeutic index. Given the substantial prevalence of *ATM* alterations and *TP53* mutations in gastric cancer, these findings highlight ATR inhibition as a promising synthetic lethal approach that may exploit context-specific vulnerabilities in gastric cancer. Continued clinical evaluation of ATRis, particularly in biomarker-enriched gastric cancer populations, could refine precision-based strategies and improve outcomes in this challenging malignancy [65,119,127,128,129].

Furthermore, gastric tumors with MSI-high status and/or AT-rich interaction domain 1A (*ARID1A*) loss, molecular subtypes enriched in certain gastric cancer subsets, exhibit impaired HR and defective G2/M checkpoint control. These alterations render tumor cells highly dependent on ATR for genomic stability, and thus exceptionally sensitive to ATR inhibition. Indeed, *ARID1A*-deficient gastric cancer cells have shown pronounced genomic instability and apoptotic cell death following ATR inhibition, both in vitro and in patient-derived organoid models. Further studies are needed to clarify the utility of RS markers and tumor suppressor deficiencies, such as *TP53* loss or *MYC* overexpression, as predictive biomarkers for ATR inhibitor monotherapy in gastric cancer. While these features have been associated with increased sensitivity to ATR inhibition in other tumor types, their specific role in gastric cancer remains uncharacterized. This knowledge gap is particularly relevant given the emerging therapeutic potential of ATRis beyond HRD tumors, especially in the context of treatment resistance. Notably, *ARID1A* mutations, frequently observed in gastric cancer and particularly enriched in the TCGA genomically stable and Epstein–Barr virus (EBV)-positive subtypes, have been linked to altered chromatin remodeling, impaired DNA repair, and increased RS, molecular hallmarks that may confer heightened vulnerability to ATR blockade. Immunohistochemical assessment of ARID1A loss has also shown prognostic value, correlating with poor differentiation, lymph node involvement, and resistance to standard therapies such as platinum-based chemotherapy. In addition, *ARID1A* deficiency has been implicated in modulating response to immunotherapy, PARP inhibitors, and epigenetic therapies, thereby positioning it as a central biomarker for patient stratification. Given the potential for ATRis to serve as a salvage strategy for patients progressing on PARP inhibitors, particularly in the *ARID1A*-deficient or HR-proficient gastric cancer subgroups, integrating *ARID1A* testing, via IHC or genomic profiling, into clinical workflows may enhance therapeutic precision. Ultimately, prospective studies are warranted to determine whether co-existing *ARID1A* loss, TP53 dysfunction, or oncogene-induced RS can guide the clinical deployment of ATRis either as monotherapy or in rational combination regimens within the gastric cancer landscape [119,124,130,131,132].

This synthetic vulnerability of cancer cells, particularly those already experiencing high levels of RS or defective DDR pathways, underpins the therapeutic rationale for ATR inhibitor monotherapy or combination regimens that further destabilize replication processes. Preclinical genetic screens have also revealed numerous other synthetic lethal interactions with ATRi, including those involving *ARID1A*, ribonuclease H2 subunit A/B (*RNASEH2A/B*), DNA polymerase epsilon 3/4, accessory subunit (*POLE3/4*), and apolipoprotein B mRNA editing enzyme, catalytic polypeptide-like 3A/B (*APOBEC3A/B*). Conversely, resistance to ATRi has also been observed through loss or mutation of key RS regulators such as cyclin E (*CCNE1*), *CDK2*, *MYC*, and *CDC25A/B*, or through cell cycle arrest mechanisms mediated by forkhead box M1 (*FOXM1*), *CDK8/Cyclin C*, and epithelial cell transforming 2 oncogene (*ECT2*). Additionally, the emergence of *ATR* mutations that reduce drug binding while preserving catalytic activity represents a plausible resistance mechanism, similar to resistance pathways seen with PARP inhibitors. However, unlike revertible tumor suppressors such as *BRCA1/2*, some ATRi synthetic lethal partners (e.g., *ARID1A*) may be essential for tumor fitness, potentially limiting reversion-based resistance. These insights suggest that the durability of ATRi response may depend on whether a tumor’s genetic vulnerabilities are transiently or permanently required for its survival. Moreover, emerging evidence highlights that S/G2 or G2/M cell cycle arrest can confer resistance to ATRi by facilitating recovery from RS. Thus, rational combination strategies that avoid antagonistic interactions, such as co-administration with CDK4/6 inhibitors, may be necessary to optimize the clinical efficacy of ATR inhibition (ATRi). This topic was discussed by Baxter et al. [133].

Emerging data from genome-wide CRISPR–Cas9 screens have elucidated multiple resistance mechanisms to ATRi, offering critical insights into the determinants of ATRi efficacy across distinct genomic landscapes. For example, in both *ATM*-proficient and *ATM*-deficient contexts, loss-of-function mutations in cyclin C (*CCNC*) and *CDK8*, key components of the CDK8 kinase module of the RNA polymerase II mediator complex, conferred significant resistance to ATR and CHK1 inhibition. Mechanistically, this resistance is not mediated through canonical checkpoint regulators such as CDC25A but rather through the suppression of transcription-associated RS. Specifically, *CCNC* or *CDK8* deficiency reduces basal and ATRi-induced DNA:RNA hybrid (R-loop) formation during S-phase, consequently mitigating transcription–replication collisions and replication fork collapse, which are pivotal triggers of ATRi-induced cytotoxicity. This reduction in RS translates into decreased micronuclei formation and delays in replication catastrophe, ultimately promoting cell survival even in the face of RS response pathway inhibition. Interestingly, this protective phenotype was phenocopied by transcriptional inhibition but not by RNase H1 overexpression, supporting a transcription-dependent mechanism. Importantly, these resistance mechanisms were operative in both ATM WT and KO cells, underscoring their potential universality and clinical relevance. In contrast, few resistance-conferring gene deletions were identified in *ATM*-deficient cells, suggesting that ATR inhibition in this context triggers cell death through multiple, non-redundant pathways, making resistance more difficult to establish. The identification of YTH N6-methyladenosine RNA binding protein 2 (*YTHDF2*) and *RECQL5*, regulators of R-loop stability and transcription–replication conflict resolution, as sensitizers to ATRi further reinforces the role of transcription-coupled RS in determining therapeutic response. Together, these findings emphasize that transcription-associated RS is a modifiable determinant of ATRi sensitivity and may represent both a vulnerability and a resistance mechanism in tumors. Thus, assessing basal DNA:RNA hybrid levels and transcription-replication dynamics may provide predictive biomarkers for ATRi responsiveness, while also informing rational combination therapies that preempt or overcome resistance [134].

Furthermore, O’Leary et al. found that the loss of UP-frameshift protein 2 (UPF2) and other components of the nonsense-mediated mRNA decay (NMD) pathway may constitute a route through which cancer cells evade ATRi-induced cytotoxicity. This resistance phenotype was consistent across multiple gastric cancer models, including AGS, YCC6, and HGC27 cell lines, and extended beyond ATR inhibition to encompass CHK1 and G2 checkpoint kinase (WEE1) inhibitors, underscoring the critical role of NMD factors in mediating the broader RS response. Proteomic, phosphoproteomic, and transcriptomic profiling of *UPF2*-deficient cells revealed a pronounced suppression of DDR signaling and impaired activation of the G1/S checkpoint upon ATRi treatment. In contrast to parental cells, which triggered ATM-CHK2 activation in response to ATRi, *UPF2*-knockout cells progressed through the cell cycle, suggesting that UPF2 is necessary for ATM-mediated checkpoint engagement under RS conditions. Notably, while UPF1 has been linked to R-loop formation, ectopic expression of RNase H1 failed to reverse ATRi resistance in *UPF2*-deficient cells, indicating that R-loops are not the primary driver of this resistance mechanism. Instead, reduced transcription-replication conflicts (TRCs) and diminished replication fork stalling were observed in *UPF2*-deficient cells, leading to decreased dependence on ATR for fork stabilization and recovery. These findings parallel earlier observations with *CNCC* and *CDK8* loss, highlighting a broader paradigm wherein attenuation of TRCs suppresses ATRi sensitivity. Taken together, this study reveals a previously unrecognized function of UPF2 in regulating TRC-associated RS and proposes NMD deficiency as a biomarker of ATRi resistance. Consequently, assessing NMD pathway integrity may aid in stratifying gastric cancer patients likely to benefit from ATR-targeted therapies, while simultaneously guiding combination strategies to circumvent resistance [135].

Similarly, resistance to CHK1 inhibitors poses a significant barrier to their effective clinical application, especially in cancers characterized by elevated RS. Despite initial sensitivity, tumor cells frequently develop resistance through diverse and often convergent mechanisms. In Eµ-MYC-driven B-cell lymphomas, for example, perturbation of the nuclear factor kappa-light-chain-enhancer of activated B cells (NF-κB) pathway through loss of cellular homolog of the viral oncogene Rel (*cREL*) or mutation of RELA proto-oncogene, NF-kB subunit (*RELA*) (T505A) leads to CHK1 inhibitor resistance, either by complete loss of CHK1 protein, driven by ubiquitin carboxyl-terminal hydrolase 1 (USP1) down-regulation, or by reduced CHK1 activity due to diminished CLASPIN expression. These modifications prevent effective CHK1 targeting, highlighting the centrality of upstream regulatory networks in modulating drug response. Moreover, cells adapt to the loss of CHK1 signaling by activating compensatory survival pathways such as phosphoinositide 3-kinase/ protein kinase B (PI3K/PKB) or Ras homologous (RHO) small GTPase/Ras-related C3 botulinum toxin substrate (RAC) small GTPase/P21-activated kinase (RHO/RAC/PAK), which support continued proliferation and resistance to apoptosis under RS. Similarly, in small cell lung cancer (SCLC), acquired resistance to the CHK1 inhibitor prexasertib correlates with increased expression of WEE1, another cell cycle regulator. Inhibition or knockdown of WEE1 can reverse this resistance, suggesting a synthetic vulnerability that may be therapeutically exploited. Additionally, bypass activation of signaling pathways such as p38 mitogen-activated protein kinase (P38MAPK) further contributes to resistance, underscoring the multifactorial nature of the adaptive response. Resistance is also associated with altered DNA damage repair dynamics, modulation of protein synthesis, and differential activation of factors like AMP-activated protein kinase (AMPK) and MUS81. Collectively, these studies reveal that CHK1 inhibitor resistance arises through a combination of protein loss, functional inactivation, and cellular rewiring of pro-survival networks. Understanding the molecular underpinnings of these resistance mechanisms will be critical to improving the therapeutic efficacy of CHK1 inhibitors and developing rational combination strategies to overcome or prevent resistance, as discussed by other authors [136,137,138].

Furthermore, as with other anti-cancer agents used in clinical settings, both ATR and CHK1 inhibitors are associated with limiting side effects that can impact treatment feasibility and patient outcomes. One of the most common and clinically significant adverse effects of ATR inhibition is reversible anemia, which appears to be a class effect [139,140,141]. This toxicity is primarily attributed to the heightened sensitivity of erythroblast precursors to iron-dependent ROS, rendering them exceptionally vulnerable to ATR inhibition through a mechanism involving enhanced ferroptosis [142]. Consequently, ATRis suppress erythroblast proliferation and differentiation in a dose-dependent manner, leading to a reduction in reticulocyte numbers and the onset of anemia. Importantly, this side effect can be mitigated by adjusting the dosing regimen. Intermittent dosing schedules, such as 4-days-on/3-days-off or 1-week-on/2-weeks-off, have been shown to reduce the severity and incidence of anemia and broader myelosuppression while maintaining effective target inhibition. These alternative dosing strategies allow time for erythroid precursor maturation and hematologic recovery, thereby enhancing tolerability. However, optimal scheduling must be carefully determined through preclinical modeling and may require customization based on the specific ATR inhibitor used. This is particularly crucial when designing combination regimens, where alternating schedules may help to minimize overlapping toxicities [143].

Similarly, the design and development of CHK1 inhibitors present multiple challenges related to biomarker validation, selectivity, and safety. Reliable pharmacodynamic assessment remains difficult due to the limited availability of dynamic and noninvasive biomarkers. Although immunohistochemical analyses of markers such as phosphorylated histone H3, γH2AX, and phosphorylated CDK1 have been employed to monitor CHK1 pathway activity, these assays require serial tumor biopsies, which are often not feasible in clinical settings. Moreover, the paradoxical increase in CHK1 phosphorylation at Ser317 and Ser345 following inhibitor treatment complicates pharmacodynamic interpretation, likely reflecting feedback loops involving ATR rather than effective target suppression. More specific markers, such as CHK1 autophosphorylation at Ser296, may offer greater fidelity in assessing target engagement. Noninvasive imaging modalities, such as 3′-deoxy-3′-[^18^F]fluorothymidine positron emission tomography (FLT-PET), have shown promise in preclinical models and could serve as valuable tools for real-time monitoring of CHK1 inhibitor activity in future trials. In addition to biomarker limitations, concerns remain regarding the long-term safety and specificity of CHK1 inhibition. CHK1 is essential for maintaining replication fork stability and genome integrity, even in unperturbed cells, and its chronic inhibition may have deleterious effects on normal proliferative tissues. Although newer agents have improved selectivity, most remain ATP-competitive and may inhibit other ATP-utilizing kinases or enzymes, potentially contributing to off-target toxicities, including cardiotoxicity. These issues underscore the need for more selective drug designs, such as allosteric inhibitors, which may mitigate off-target effects while preserving on-target activity. Together, these factors highlight the importance of continued refinement in both CHK1 inhibitor chemistry and clinical trial design to fully realize the therapeutic potential of this class [144].

**Figure 4:**
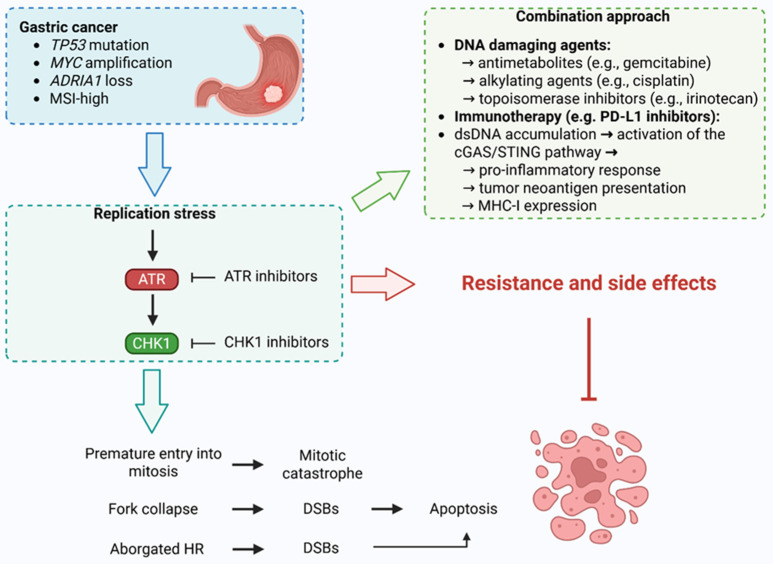
Framework for the clinical application of ATR–CHK1 axis inhibitors in gastric cancer. Integration of context-specific molecular vulnerabilities, such as *TP53* mutations, *ARID1A* loss, *ATM* deficiency, and oncogene-driven replication stress, enables selective targeting of tumor cells while sparing normal tissue. Resistance mechanisms, including modulation of transcription-associated replication stress and activation of bypass survival pathways, inform the design of rational combination therapies to enhance efficacy and prevent therapeutic escape. Treatment optimization strategies incorporating biomarker-guided patient selection, adaptive dosing to reduce hematologic toxicity, and refined pharmacodynamic monitoring support the safe and effective clinical implementation of ATR–CHK1 inhibitors in biomarker-defined subsets of gastric cancer. ARID1A—AT-rich interaction domain 1A; ATR—ataxia telangiectasia and Rad3-related protein kinase; CHK1—checkpoint kinase 1; DSBs—DNA double-strand breaks; HR—homologous recombination; MHC—major histocompatibility complex; MSI—microsatellite instability; MYC—myelocytomatosis oncogene; PD-L1—programmed death-ligand 1. Created in BioRender. Kciuk, M. (2025) https://BioRender.com/3w2f04f (accessed on 8 August 2025).

In summary, integrating the diverse mechanistic insights and molecular vulnerabilities outlined above provides a compelling framework for the efficient and safe clinical application of ATR–CHK1 axis inhibitors in gastric cancer. By identifying and targeting context-specific genetic alterations, such as *TP53* mutations, *ARID1A* loss, *ATM* deficiency, and oncogene-driven RS, clinicians can exploit tumor-specific dependencies while sparing normal cells. Moreover, understanding resistance mechanisms, including transcription-associated RS modulation and bypass survival pathways, enables the design of rational combination regimens that both enhance efficacy and mitigate resistance. Importantly, optimizing treatment through biomarker-guided patient selection, adaptive dosing strategies to limit hematologic toxicity (e.g., anemia), and refined pharmacodynamic monitoring can further enhance tolerability and therapeutic precision. Collectively, these approaches support the advancement of ATR–CHK1 inhibitors from experimental agents to clinically viable tools for overcoming resistance and improving outcomes in biomarker-defined subsets of gastric cancer (Figure 4).

## Figures and Tables

**Table 1:** Preclinical and clinical studies investigating ATR inhibitors for gastric cancer treatment.

Study/Trial	Focus	Model/System	Key Findings	Mechanism/Pathway	Clinical Relevance	PMID
VE-821 and Cisplatin in Gastric Cancer	Effect of ATR inhibitor VE-821 on cisplatin sensitivity	Gastric cancer cell lines (MKN-45, AGS), cell organoids, in vivo models	VE-821 inhibits proliferation, induces apoptosis, blocks ATR/CHK1 phosphorylation, reverses STAT3 activation, enhances DNA damage, synergizes with cisplatin	ATR-mediated DDR pathway inhibition; STAT3 signaling reversal	Higher ATR expression correlates with advanced gastric cancer stages; VE-821 enhances cisplatin sensitivity	37517142
PAK6 and Oxaliplatin Resistance	Role of PAK6 in DDR and chemoresistance to oxaliplatin	Gastric cancer clinical samples and cells	PAK6 promotes chemoresistance by activating ATR → CHK1 → RAD51 pathway; blocking ATR with AZD6738 reverses resistance	PAK6 → ATR → CHK1 → RAD51 HR repair pathway	PAK6 expression correlates with aggressive disease and oxaliplatin resistance; ATR inhibition overcomes resistance	35902562
AZD6738 in ATM-deficient Gastric Cancer	ATR inhibition efficacy in ATM-deficient models	Gastric cancer cell lines (SNU-601 ATM-deficient, SNU-484 ATM-intact), xenografts	AZD6738 causes DNA damage accumulation, cell cycle arrest, apoptosis in ATM-deficient cells; ATM inhibition sensitizes ATM-intact cells	Synthetic lethality: ATR inhibition + ATM deficiency; possible HDAC1 involvement in sensitivity	Supports biomarker-driven trials targeting ATR in ATM-deficient gastric cancers	28138034
Phase II Clinical Trial (NCT03780608)	AZD6738 + durvalumab in advanced gastric cancer (AGC)	Patients with previously treated AGC	Combination showed ORR 22.6%, disease control rate 58.1%, manageable toxicity; ATM loss or HR deficiency biomarkers predict better PFS; immune activation seen in responders	ATR inhibition → cGAS-STING pathway activation → enhanced anti-tumor immunity; checkpoint blockade synergy	DDR alterations are predictive biomarkers; supports DDR-immune checkpoint inhibitor combos in gastric cancer	35790315

AGC—advanced gastric cancer; ATR—ataxia telangiectasia and Rad3-related protein kinase; AZD6738—ceralasertib (ATR inhibitor); CHK1—checkpoint kinase 1; DDR—DNA damage response; HR—homologous recombination; HDAC1—histone deacetylase 1; ORR—objective response rate; PAK6—p21 (RAC1) activated kinase 6; PFS—progression-free survival; RAD51—RADiation sensitive 51; STAT3—signal transducer and activator of transcription 3; VE-821—selective ATR kinase inhibitor; cGAS—cyclic GMP–AMP synthase; STING—stimulator of interferon genes.

**Table 2:** Clinical trials investigating ATR inhibitors for cancer treatment.

Trial ID	Phase	Drug(s) Tested	Combination(s)	Cancer Type(s)	Objectives/Endpoints	Design Features	Key Biomarkers/Scientific Rationale
NCT02264678	Phase I/1b	AZD6738	Carboplatin (Module 1), Olaparib (Module 2), Durvalumab (Module 3), AZD5305 (Module 5)	Advanced/metastatic solid tumors, including gastric cancer	Safety, tolerability, pharmacokinetics, preliminary efficacy	Modular design, dose-escalation (Part A), cohort expansion (Part B), food effect and QT interval module	ATR inhibition disrupts DNA repair; synthetic lethality with platinum-based and PARP inhibitors; immunomodulatory effect with durvalumab; selective DDR targeting with AZD5305
NCT04704661	Phase I/Ib	AZD6738 + trastuzumab deruxtecan (DS-8201a)	Combination vs. monotherapy in dose-expansion phase	Advanced solid tumors with HER2 alterations	Safety, pharmacodynamics, preliminary efficacy; recommended Phase II dose; tolerability	Dose-escalation and dose-expansion; run-in monotherapy cycle; pharmacodynamic biomarker analysis; biorepository	HER2-targeting ADC causing DSBs + ATR inhibition blocking DDR; biomarkers include phosphorylated RAD50, SLFN11, HER2 heterogeneity, TP53, ATM, RAS mutations
NCT03641313	Phase II	Berzosertib (M6620) + irinotecan	Combination	Metastatic/unresectable gastric/GEJ cancer, TP53-mutant focus	Improvement in ORR vs. historical control; secondary: PFS, OS, DOR, TTP	Biomarker-driven trial focusing on TP53 mutation; correlative biomarker analyses	TP53 mutations increase reliance on ATR G2/M checkpoint; synthetic lethality exploited by ATR inhibitor berzosertib
NCT04535401	Phase I	Elimusertib (BAY 1895344) + FOLFIRI	Combination	Gastrointestinal malignancies	Determine MTD; secondary: ORR, PFS, OS, clinical benefit rate; pharmacokinetics; biomarker analyses	Dose-escalation + cohort expansion; paired biopsies and serial imaging	ATM pathway inhibition combined with chemotherapy; exploratory: UGT1A1 genotype, tumor mutation profiles, ATM expression
NCT04491942	Phase I	Elimusertib + cisplatin +/− gemcitabine	Doublet: cisplatin + elimusertib; Triplet: cisplatin + gemcitabine + elimusertib	Advanced solid tumors, incl. metastatic urothelial carcinoma (UC)	Establish MTD, recommended Phase II dose; safety; pharmacokinetics; preliminary anti-tumor activity	Dose-escalation; two treatment arms	Biomarkers of DDR deficiency; somatic gene alterations; circulating tumor DNA (ctDNA); comprehensive genomic/transcriptomic profiling

ADC—antibody–drug conjugate; AGC—advanced gastric cancer; ATM—ataxia telangiectasia mutated; ATR—ataxia telangiectasia and Rad3-related protein kinase; ctDNA—circulating tumor DNA; DDR—DNA damage response; DSB—double-strand break; DOR—duration of response; FOLFIRI—folinic acid, fluorouracil, and irinotecan; GEJ—gastroesophageal junction; HER2—human epidermal growth factor receptor 2; MTD—maximum tolerated dose; ORR—objective response rate; OS—overall survival; PARP—poly (ADP-ribose) polymerase; PFS—progression-free survival; RAS—rat sarcoma viral oncogene homolog; SLFN11—schlafen family member 11; TP53—tumor protein p53; TTP—time to progression; UGT1A1—UDP-glucuronosyltransferase family 1 member A1; UC—urothelial carcinoma.

## Abbreviations

The following abbreviations are used in this manuscript:

AMPK	AMP-activated protein kinase
ARID1A	AT-rich interaction domain 1A
ATM	ataxia-telangiectasia mutated
ATR	ataxia telangiectasia and Rad3-related protein kinase
ATRIP	ATR-interacting protein
ATRis	ATR inhibitors
BAK	BCL2 antagonist/killer 1
BAX	BCL2 associated X
BRCA1/2	breast cancer type 1 susceptibility protein 1/2
BRIP1	BRCA1 interacting protein C-terminal helicase 1
CAD	caspase-activated Dnase
CASPASE5	cysteine-aspartic acid protease 5
CCNC	cyclin C
CCNE1	cyclin E
CDC25A/25C/45	cell division cycle 25A/25C/45
CDH1	cadherin 1
CDK1/2	cyclin-dependent kinases 1/2
CDR	coding region
cGAS-STING	cyclic GMP-AMP synthase-stimulator of interferon genes
CHK1/2	checkpoint kinases 1/2
ctDNA	circulating tumor DNA
DCR	disease control rate
DDK	DBF4-dependent kinase
DDR	DNA damage response
DNA-PKcs	DNA-dependent protein kinase catalytic subunit
DOR	duration of response
DSB	DNA double-strand break
DSBR	DNA double-strand break repair
EBV	Epstein–Barr virus
ecDNA	extrachromosomal DNA
ECT2	epithelial cell transforming 2 oncogene
EGFR	epidermal growth factor receptor
ERBB2	erb-b2 receptor tyrosine kinase 2
ETAA1	Ewing’s tumor-associated antigen 1
FANCC	fanconi anemia complementation group C
FAS	fas cell surface death receptor
FGFR2	fibroblast growth factor receptor 2
FLT-PET	3′-deoxy-3′-[^18^F]fluorothymidine positron emission tomo
FOLFIRI	leucovorin calcium, fluorouracil, and irinotecan hydrochloride
FOXM1	forkhead box M1
GEJ	gastric and gastroesophageal junction
HDAC1	histone deacetylase 1
HDGC	hereditary diffuse gastric cancer
HER2	human epidermal growth factor receptor 2
hMLH1	mutL homolog 1
hMSH3	mutS homolog 3
hMSH6	mutS homolog 6
HR	homologous recombination
HRD	homologous recombination deficiency
IARC	International Agency for Research on Cancer
IGFIIR	insulin-like growth factor II receptor
ILF2	interleukin enhancer binding factor 2
KU70	X-ray repair cross-complementing 6
MDC1	mediator of DNA damage checkpoint 1
MDM2	E3 ubiquitin-protein ligase
MED1	mediator complex subunit 1
MHC	major histocompatibility complex
MLL	mixed-lineage leukemia protein
MMR	mismatch repair
MRE11	meiotic recombination 11 homolog A
MSH2	mutS homolog 2
MSI	microsatellite instability
MTD	maximum tolerated dose
MYC	myelocytomatosis oncogene
NBS1	Nijmegen breakage syndrome protein 1
NF-κB	nuclear factor kappa-light-chain-enhancer of activated B cells
NGS	next-generation sequencing
NMD	nonsense-mediated mRNA decay
NOXA	phorbol-12-myristate-13-acetate-induced protein 1
ORC	origin recognition complex
ORR	overall response rate
P38MAPK	p38 mitogen-activated protein kinase
PAK6	p21 (RAC1) activated kinase 6
PALB2	partner and localizer of BRCA2
PARP	poly (ADP-ribose) polymerase
PBMCs	peripheral blood mononuclear cells
PD-L1	programmed death-ligand 1
PDX	patient-derived xenograft
PFS	progression-free survival
PI3K	phosphoinositide 3-kinase
PIK3CA	phosphatidylinositol-4,5-bisphosphate 3-kinase catalytic subunit alpha
PKB	protein kinase B
pRB	retinoblastoma protein
PUMA	BCL2 binding component 3
RAC	Ras-related C3 botulinum toxin substrate
RAD50	RADiation sensitive 50
RAD51	RADiation sensitive 51
RAS	rat sarcoma viral oncogene homolog
RECQL5	RecQ-like helicase 5
RHO	ras homologous
ROS	reactive oxygen species
RPA	replication protein A
RS	replication stress
SCLC	small cell lung cancer
shRNA	short hairpin RNA
SLFN11	schlafen family member 11
ssDNA	single-stranded DNA
STAT3	signal transducer and activator of transcription 3
T14	threonine 14
TCF4	T-cell factor 4
TCGA	The Cancer Genome Atlas
TEAE	treatment-emergent adverse event
TGFβRII	transforming growth factor beta receptor type II
TOPBP1	topoisomerase 2-binding protein 1
TP53	tumor protein p53
TTP	time to progression
UC	urothelial carcinoma
UGT1A1	uridine 5′-diphospho-glucuronosyltransferase family 1 member A1
UPF2	UP-frameshift protein 2
USP1	ubiquitin carboxyl-terminal hydrolase 1
WEE1	G2 checkpoint kinase
Y15/17/24	tyrosine 15/17/24
YTHDF2	YTH N6-methyladenosine RNA binding protein 2
γH2AX	phosphorylated histone H2AX on serine 139
